# Ionizing Irradiation Regulation of Rice Product Quality: A Comprehensive Analysis Based on Starch Multiscale Structure

**DOI:** 10.3390/foods15152681

**Published:** 2026-07-29

**Authors:** Bin Ren, Danyang Liang, Yuheng Zhai, Ying Wan, Xiaohu Luo

**Affiliations:** 1 College of Food Science and Engineering, Lingnan Normal University, Zhanjiang 524048, China; 2 Zhejiang-Malaysia Joint Research Laboratory for Agricultural Product Processing and Nutrition, College of Food Science and Engineering, Ningbo University, Ningbo 315832, China

**Keywords:** ionizing irradiation, rice starch, multiscale structure, quality regulation, structure-quality relationship

## Abstract

Rice and rice-derived products are staple foods worldwide, creating an urgent demand for green processing technologies that ensure safety, preserve quality, and extend shelf life. Ionizing irradiation, characterized by high efficiency, non-thermal processing, and the absence of chemical residues, has been increasingly utilized in the storage, preservation, and quality control of rice and rice-based products. As the principal component of rice, starch exhibits a multiscale structural organization spanning molecular to macroscopic levels, which fundamentally determines cooking performance, eating quality, and storage stability. Through modulation of the multiscale structure of starch, ionizing irradiation can achieve targeted quality improvement and stabilization of rice products. This review summarizes the effects of three common types of ionizing irradiation on the structural evolution of rice starch. With a focus on practical applications, it further elucidates the intrinsic relationships between irradiation-induced starch structural modifications and the physicochemical properties, sensory quality, and storage stability of rice products. In addition, current industrial applications and existing technical limitations are critically discussed, and targeted optimization strategies. Finally, based on the fundamental mechanisms underlying starch “structure–quality” regulation, future application-oriented research directions are highlighted.

## 1. Introduction

Rice is one of the world’s major food crops, with a long history of cultivation and processing [1]. With the advancement of the modern food industry and the growing emphasis on nutrition, rice is no longer confined to its traditional role as a staple food. Owing to its mild flavor, low allergenicity, and high digestibility, rice has become an increasingly important ingredient in gluten-free food products [2]. In addition to refined packaged rice and rice starch, a wide variety of rice-based products, including fresh rice noodles [3], dried rice noodles [4], rice steamed bread [5], rice bread [6], instant rice [7], beverages [8], and rice puddings [9], have greatly expanded the diversity of rice-derived foods. Rice is characterized by high annual production and a stable harvest cycle [10], which has driven continuous efforts to develop effective post-harvest preservation technologies to maintain its edible quality and processing performance from harvest through industrial processing. Specifically, the physicochemical properties and quality attributes of rice serve not only as key indicators for selecting appropriate storage methods, but also as important criteria for evaluating its suitability as a food-processing raw material. Therefore, the development of safe, efficient, and environmentally friendly storage and quality-control technologies is of great significance for ensuring food security and improving the quality of rice processing.

Against this background, irradiation technology has become a major focus of research and application in food processing because of its notable advantages, including non-thermal treatment, high efficiency, and the absence of chemical residues. Ionizing irradiation shows considerable potential for application in the modern food industry. According to the primary radiation source, ionizing irradiation can be categorized into γ-rays, electron beam irradiation (EBI), and X-ray irradiation. Among these, γ-rays are generated through the spontaneous decay of the radioactive nuclide ^60^Co, electron beams are produced by directly accelerating electrons using an accelerator, and X-rays are generated when accelerated electron beams bombard heavy metal targets through bremsstrahlung radiation [11]. Due to its aforementioned advantages, ionizing irradiation has been widely applied in food microbial control [12], sterilization of food packaging materials [13], preservation of fresh agricultural products [14], and modification of specific functional components [15]. In addition, ionizing irradiation technology has been extensively utilized in grain processing. Compared with conventional preservation methods, irradiation enables effective control of microbial contamination during the storage of high-moisture rice [16,17], while avoiding significant losses of the major nutritional components of rice [18,19], significantly extends storage life.

However, its effects on the internal constituents of the food matrix cannot be overlooked, even within the permissible safety dose range for food applications [11].

Starch is the principal component of rice, and its functional properties are jointly determined by amylose and amylopectin [20]. The amylose/amylopectin ratio varies among rice cultivars. Based on their amylopectin content, these varieties can be categorized into three groups: waxy rice (with the highest amylopectin content), japonica rice (intermediate), and indica rice (the lowest). At the molecular level, both amylose and amylopectin exhibit characteristic multiscale structural features, including the molecular scale represented by average molecular weight and chain length distribution, the supramolecular scale involving double-helix assembly and crystalline-region formation, the microscopic scale associated with starch granule morphology and internal aggregation state [21], and the macroscopic scale ultimately reflected in processing performance and eating quality. The multiscale structural characteristics of starch molecules, including chain length, crystallinity, ordered structure, and gel network formation, directly determine the gelatinization behavior, retrogradation properties, rheological characteristics, and texture and sensory quality of rice products [22,23]. In other words, apart from components such as starch, lipids and water, the molecular structure of starch is one of the most important intrinsic factor governing the quality of processed rice products. Ionizing irradiation can regulate the multiscale structure of starch through mechanisms such as glycosidic bond cleavage, crystalline structure disruption, and alteration of molecular aggregation states [11]. These structural modifications are subsequently translated into changes in functional properties, ultimately influencing the quality formation and storage stability of rice products [16].

Based on these, this narrative review focuses on the multiscale structure of rice starch and provides a comprehensive overview of irradiation-induced changes in its physicochemical and functional properties and their consequent effects on product quality, rather than adhering to a rigid systematic review protocol. By integrating current studies on rice and rice starch, this review further discusses the regulatory effects of three common ionizing irradiation methods on the quality of rice products, aiming to offer theoretical support and practical guidance for the industrial application of irradiation technology in rice processing and for raw material selection and process optimization. To ensure transparency in the literature assembly process, we systematically searched Scopus and Web of Science using a combination of keywords, including “ionizing radiation”, “gamma irradiation”, “electron beam”, “X-ray”, “starch”, “rice starch”, “starch multiscale structure”, “starch physicochemical properties”, “rice quality”, and “food irradiation”. We limited our search to peer-reviewed journal articles published in English, with an emphasis on works from 2020 to 2026. Duplicate records and non-English publications were excluded. In addition to the database search, we employed a pearl-fishing strategy by manually screening the reference lists of articles highly relevant to our core topic and tracking recent publications that cited older foundational studies. During screening, we first evaluated titles and abstracts for relevance, specifically targeting studies that explicitly examined the interplay between irradiation parameters and starch structural hierarchies. Potentially eligible articles were then read in full, and those focusing on rice starch and rice eating quality were selected for further integration. From these, we extracted key data on irradiation sources, dose regimes, starch structural alterations at molecular, supramolecular, and microscopic scales, and corresponding physicochemical or processing properties. Through iterative cross-referencing and full-text analysis, additional relevant studies were identified and incorporated. The extracted information was thematically categorized by irradiation type and starch structural level, enabling us to map the structure-quality relationships that form the basis of this review.

## 2. Ionizing Irradiation in the Food Industry

As an emerging food processing technology characterized by short wavelengths and high energy, ionizing irradiation can remove outer-shell electrons from atoms to generate ions without causing atomic decomposition or leaving radioactive residues in the treated substrate, thereby ensuring a high level of application safety [24]. The effects of ionizing irradiation on food components are mainly achieved through two mechanisms: direct action and indirect action. Direct action refers to the direct interaction of irradiation energy with biological macromolecules in food systems, such as carbohydrates, DNA, and lipids, leading to structural damage at the molecular level. Indirect action is more common in fresh agricultural products with high moisture content, where irradiation induces the radiolysis of water molecules, generating reactive species such as hydroxyl radicals and hydrated electrons [25]. These reactive intermediates subsequently trigger chain reactions that indirectly affect food components or microorganisms [11,26].

Currently, the most widely applied forms of ionizing irradiation in the food industry are γ-rays, EBI, and X-ray irradiation. Within the permissible irradiation dose range, all three technologies can effectively regulate target components in food systems [27]. In addition to ionizing irradiation, non-ionizing irradiation technologies, such as ultraviolet irradiation, are also commonly used in food processing. However, due to the scope and central focus of this review, these technologies will not be discussed in detail.

### 2.1. γ-Ray

γ-rays are characterized by high energy, long propagation distances in air, and strong penetration capability [28]. They can deeply penetrate food matrices and achieve efficient sterilization by disrupting the covalent bonds within microbial DNA. Consequently, γ-rays are widely used for the sterilization of bulk and packaged foods [28,29].

The irradiation energy of γ-rays originates from atomic nuclei, and their emission primarily relies on two radioactive isotopes, ^60^Co and ^137^Cs [30]. Dependence on radioactive isotopes is the fundamental factor limiting the broader application of γ-ray technology. Among the available radiation sources, ^60^Co is generally preferred for food irradiation because of its low water solubility and relatively lower environmental contamination risk. In contrast, ^137^Cs is highly water-soluble and poses a substantially greater risk of environmental pollution, resulting in much stricter limitations on its application [31]. In addition, γ-rays inherently involve risks associated with radioactive decay [30], and these safety concerns have contributed to persistently negative consumer perceptions of γ-ray technology [32].

Due to the strong dependence of γ-rays on radioactive isotopes, together with the associated environmental and safety concerns, its application scale in the food processing industry has gradually declined in recent years. Nevertheless, γ-ray remains one of the ionizing radiation technologies that have been studied for rice processing, insights derived from these studies continue to offer useful mechanistic references.

### 2.2. X-Ray

The strong penetration capability of X-rays has been extensively demonstrated in the field of medical imaging, and this property has also proven highly effective in food processing applications. X-rays can generally penetrate food matrices with thicknesses of approximately 30~40 cm, making them highly suitable for food irradiation treatments [33].

The energy of X-rays originates from electronic transitions outside the atomic nucleus and is generated when high-energy accelerated electrons bombard target materials with high atomic numbers [30]. The photons produced during these electronic transitions serve as the primary carriers of energy transfer, and their energy is distributed across a continuous spectrum [34].

Compared with γ-rays, the generation of X-rays does not involve radioactive isotopes, providing significant advantages in terms of environmental friendliness and radiation safety [35]. In terms of irradiation efficiency, X-rays operated at an input energy of approximately 5 MeV can achieve penetration capabilities comparable to those of ^60^Co γ-rays [36]. Moreover, when the input energy reaches 10 MeV, X-rays can effectively reduce pathogenic microorganisms in food without introducing food safety concerns [30]. Owing to these advantages, X-ray irradiation has been widely applied in sterilization of pre-packaged foods [31], postharvest preservation of fruits and vegetables [37,38,39], and other practical food-processing applications.

However, X-ray irradiation technology also has certain limitations. The initial investment cost for X-ray equipment is relatively high, and stringent requirements are imposed on target material performance, which to some extent limits its large-scale industrial application.

### 2.3. EBI

EBI is currently one of the most widely used ionizing irradiation technologies and is widely recognized for its high level of operational safety. Similar to X-rays, EBI is generated using an electron accelerator, with its energy originating from highly accelerated electrons [11]. However, unlike X-rays, EBI does not require a target material for radiation generation, which not only improves energy conversion efficiency but also reduces equipment investment costs [35]. As a result, EBI offers greater economic advantages than X-rays in terms of equipment construction, maintenance, and processing efficiency. The maximum output energy of EBI can reach 10 MeV. As one of the most extensively applied ionizing irradiation technologies in the food industry [40], EBI has been widely used in a variety of food-processing applications, including microbial control, modification of packaging materials, preservation of fruits and vegetables, and targeted modification of food components [12,13,41,42].

The major advantages of EBI include its independence from radioactive isotopes, reduced potential hazards to operators, equipment, and the environment, high energy-utilization efficiency, and strong suitability for industrial-scale production. However, EBI also has notable limitations. Its penetration capability is relatively weak, making it difficult to achieve deep and uniform treatment of bulk foods. Consequently, EBI is more suitable for surface and shallow-layer processing applications [11].

## 3. Regulation of the Multiscale Structure of Rice Starch by Ionizing Irradiation

As the primary component governing the processing characteristics and eating quality of rice, starch exhibits a typical multiscale and hierarchical structural organization, in which structural changes are progressively transmitted across different levels. From the microscopic to the macroscopic level, rice starch can generally be classified into four hierarchical scales: molecular scale, supramolecular scale, microscopic scale, and macroscopic scale [21]. These structural levels are closely interconnected and collectively determine the processing suitability of rice raw materials as well as the final quality of rice-based products.

As an efficient and environmentally friendly physical processing technology, ionizing irradiation can initiate structural modifications at the molecular scale of starch. Through the stepwise transmission of structural effects, these changes further influence the supramolecular and microscopic scales, ultimately enabling precise regulation of the quality of rice products at the macroscopic level. Based on this framework, this section first summarizes the effects of ionizing irradiation on the multiscale structure of starch by integrating studies on starches from various sources. Building upon this foundation, the differential effects of γ-ray, X-ray irradiation, and EBI are further compared and analyzed in detail.

### 3.1. Multiscale Structural Hierarchy of Starch

**Figure 1:**
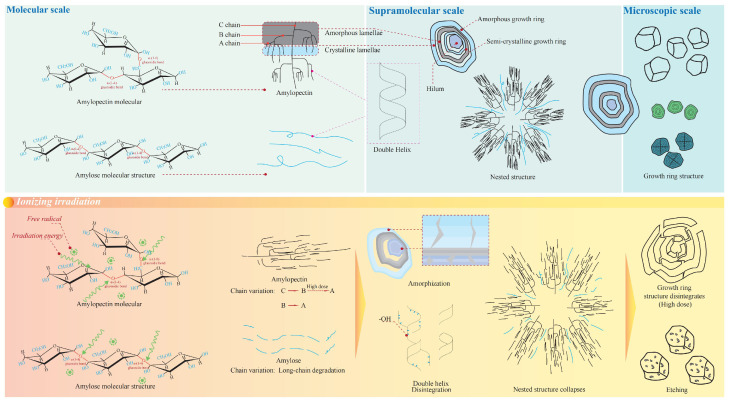
Analysis of rice starch multiscale structure and its changes induced by ionizing irradiation. (**Upper**) panel: Schematic diagram of the multiscale structure of rice starch, showing the molecular structures of amylopectin and amylose, supramolecular structure, and microscopic morphology from left to right. (**Lower**) panel: Mechanism of ionizing irradiation and the resultant multiscale structural variations of rice starch. The hierarchical structural layout corresponds sequentially to that in the upper panel.

As shown in Figure 1, at the molecular scale, rice starch, like starches from other botanical sources, is primarily composed of two major components: amylose and amylopectin [43]. The relative proportions of these two components are closely associated with rice variety. Amylose is predominantly a linear polysaccharide with a relatively high degree of polymerization, consisting mainly of glucose units linked by α-1,4 glycosidic bonds and containing only a small number of side chains [44,45]. In contrast, amylopectin possesses a highly branched structure and a much larger molecular weight, with its branch points formed through α-1,6 glycosidic linkages between glucose units. Overall, starch molecules adopt a stable left-handed helical conformation, in which glucose residues predominantly exist in the energetically favorable 4C_1_ chair conformation [46].

Based on these molecular features, starch further develops supramolecular structures. Short amylopectin branch chains assemble into double-helical units through hydrogen bonding and van der Waals interactions [21], which subsequently organize into A-type crystalline structures and periodic semicrystalline lamellae [47]. The degree of crystallinity and the integrity of the double-helical structure directly determine the proportion of ordered regions within starch granules and strongly influence their thermal stability and resistance to enzymatic digestion [48]. In addition, the distribution of hydrogen bonds both within starch molecules and between starch and water molecules plays a critical role not only in maintaining molecular structural stability, but also in regulating the hydration and gelation behaviors of starch [49,50].

At the microscopic scale, rice starch granules generally exhibit irregular polyhedral morphologies with diameters ranging from approximately 2~8 μm [51]. These granules possess a dense outer shell and an internal “onion-like” architecture consisting of alternating crystalline and semicrystalline regions [21]. In addition, channel-like structures connecting the granule interior and exterior are present within the starch granules [52]. The integrity, pore characteristics, and internal organization of starch granules at the microscopic scale directly regulate water absorption, enzymatic hydrolysis, and processing responsiveness of starch systems [53].

By integrating the structural characteristics across the molecular, supramolecular, and microscopic scales, the macroscopic scale of rice starch is typically evaluated in terms of its functional properties. Systematic multidimensional analysis of the effects of ionizing irradiation on starch structures from the molecular to the microscopic scale, together with the correlation of structural changes across different hierarchical levels, can provide important theoretical support for the application of irradiated rice and rice starch as food ingredients, as well as for evaluating their processing adaptability and product performance.

### 3.2. Effects of Ionizing Irradiation on the Multiscale Structure of Starch

Both rice starch and starches from other botanical sources exhibit highly similar multiscale assembly patterns and structural characteristics. Therefore, this section summarizes the structural response patterns of starches from different sources following ionizing irradiation treatment. The regulatory effects and mechanisms of the three major types of ionizing irradiation on starch structure are discussed from the molecular, supramolecular, and microscopic scales, respectively, together with comparative analysis based on existing studies on rice starch.

#### 3.2.1. Effects of Ionizing Irradiation on the Molecular-Scale Structure of Starch

The effects of ionizing irradiation on the molecular structure of starch can be quantitatively characterized by changes in chain-length distribution and molecular weight. On the one hand, the energy carried by ionizing irradiation primarily acts on the glycosidic bonds of starch, inducing bond cleavage and disrupting the original long-chain structures, thereby generating low-molecular-weight components [54]. On the other hand, owing to its ionizing properties, irradiation can excite or ionize surrounding water and oxygen molecules, leading to the generation of free radicals. These radicals subsequently participate in chain reactions (i.e., the indirect effects discussed previously), continuously attacking starch chains and degrading longer molecular segments into smaller fragments through glycosidic bond cleavage [26].

Considering the structural differences between amylose and amylopectin, amylose possesses a highly polymerized linear backbone with only a limited number of branch chains [44]. Due to the lack of extensive branch-chain protection, the amylose backbone is more readily exposed to the irradiation environment and therefore exhibits relatively lower structural stability [55]. In contrast, amylopectin has a highly branched and larger molecular structure. Ionizing irradiation exerts stronger debranching and molecular degradation effects on amylopectin, preferentially disrupting the outer short branch chains and thereby increasing the proportion of short chains, whereas the central backbone that forms the “tree-like” framework remains comparatively stable [56]. Such alterations in chain-length distribution generally exhibit a clear dose-dependent pattern: low irradiation doses have limited effects on short chains within crystalline regions, whereas higher doses markedly intensify long-chain degradation [11].

Meanwhile, ionizing irradiation can significantly reduce the molecular weight of starch by cleaving glycosidic bonds, disrupting the ordered structure of crystalline regions, and inducing free-radical-mediated chain reactions [35]. Similar to the dose-dependent changes observed in starch chain-length distribution, reductions in starch molecular weight are generally positively correlated with irradiation dose and cumulative irradiation exposure [35]. Although a few studies have reported weak molecular cross-linking tendencies after ionizing irradiation treatment, reflected by slight increases in apparent amylose content [57] and molecular-weight characteristics [58], the overall consensus from existing studies indicates that degradation remains the dominant effect of ionizing irradiation on the molecular weight and chain-length characteristics of most cereal, tuber, and legume starches.

#### 3.2.2. Effects of Ionizing Irradiation on the Supramolecular Structure of Starch

To understand the supramolecular structure of starch and its regulatory role in physicochemical properties, the double-helical structure should first be considered as the central structural unit. The double helix represents the fundamental building block of starch supramolecular assembly, and its stable formation generally requires at least 10 glucose residues [59]. In native starch granules, double-helical structures are primarily formed from the short branch chains of amylopectin [60,61], which subsequently undergo ordered assembly through intermolecular interactions to generate crystalline regions, semicrystalline lamellae, and periodically stacked structures distributed concentrically around the hilum of starch granules. The supramolecular structure of starch is widely regarded as the key structural bridge linking the molecular and granular scales, directly determining the thermal stability [62], gelatinization behavior [63], and retrogradation properties of starch systems [61].

Unlike NaCl, another major component commonly present in food systems that exhibits a long-range ordered ionic crystal structure throughout the entire material [64] starch is a typical semicrystalline system [65]. Its internal structure is characterized by the coexistence of crystalline and amorphous regions rather than a truly continuous single-crystal structure [66,67,68]. Within starch crystalline regions, short amylopectin branch chains first form double helices through hydrogen bonding and are then further organized into different crystalline polymorphs, including A, B, and C-type structures [68]. Rice starch, which is the main focus of this review, predominantly exhibits a typical A-type crystalline structure [69]. A-type starch crystals are characterized by relatively low crystalline water content, containing only eight water molecules per unit cell. In addition, because amylopectin branch points are distributed across both crystalline and amorphous regions and the average branch-chain length is relatively short, the crystalline lamellae of A-type starch are significantly thinner than those of B-type starch [70,71]. Owing to the presence of structurally weak points formed by internal branching within crystalline regions, A-type starch also exhibits greater sensitivity to acid hydrolysis and enzymatic degradation [71]. At present, the ordered regions of starch are commonly characterized using techniques such as FT-IR, XRD, and SAXS [11], as well as NMR [72], enabling accurate quantitative evaluation of the evolution of starch supramolecular ordering.

In previous work, we found that ionizing irradiation represented by EBI induces pronounced dose-dependent disruption of starch crystalline structures during the process of molecular fragmentation and degradation. The general trend observed is that higher irradiation doses or cumulative irradiation exposure result in lower relative crystallinity (RC) of starch [11]. This pattern has also been confirmed in starch isolated from rice following EBI treatment [19]. Notably, Lei et al. [73] reported that, compared with A-type starches, potato starch with thicker crystalline lamellae and a B-type crystalline structure exhibited significant depolymerization effects following both EBI and X-ray irradiation. Specifically, the contents of double-helical and amorphous regions increased significantly, whereas the proportion of single-helical structures decreased, leading to an overall reduction in structural order. Irradiation doses above 10 kGy increased the proportion of double helices, enhanced semicrystalline lamellar ordering and lamellar thickness, and improved structural compactness. At the same time, irradiation also increased the proportion and thickness of amorphous regions. Furthermore, the study compared the effects of the two irradiation technologies and found that high-dose X-ray irradiation exerted a more pronounced enhancement of lamellar ordering than EBI, thereby more effectively improving the digestion resistance of starch.

As summarized in Table 1, this review further integrates the reported effects of X-ray and γ-ray irradiation on the crystalline structure of A-type starches. Although EBI, X-ray irradiation, and γ-ray differ in treatment dose and energy conversion efficiency, their effects on starch structure share several common features because all three are purely physical modification technologies. Within the irradiation dose range permitted for food applications, starch generally retains the characteristic XRD diffraction peaks of A-type crystals, indicating that ionizing irradiation does not completely amorphize the semicrystalline structure of starch. In addition, FT-IR spectra do not exhibit new characteristic absorption peaks, further demonstrating that ionizing irradiation does not introduce new chemical functional groups into the starch system. Instead, irradiation-induced molecular chain scission, conformational relaxation, and moisture redistribution alter the absorption intensity and peak areas of intrinsic functional groups such as –OH, –CH–, –CH2–, and –C=O. These spectral changes are therefore not caused by the formation of new functional groups, but rather arise from the combined effects of crystalline structure disruption, molecular chain fragmentation, and rearrangement of molecular packing and vibrational environments induced by ionizing irradiation.

#### 3.2.3. Effects of Ionizing Irradiation on the Microstructural Structure of Starch

At the microscopic scale, structural changes in starch are generally evaluated based on three major features: granule surface morphology, the integrity of internal growth rings, and the preservation of the Maltese cross birefringence pattern. Fundamentally, alterations in starch microstructure can be regarded as the macroscopic manifestation of progressive structural evolution occurring at the molecular and supramolecular scales. As illustrated in Figure 1, existing studies indicate that the structural regulation of starch by ionizing irradiation originates at the molecular scale, where irradiation primarily induces starch chain degradation and molecular-weight reduction. These effects subsequently propagate to the more complex supramolecular scale, causing structural collapse of crystalline regions, reduction in crystalline order, and a significant increase in the proportion of amorphous phases [11]. Although some short-chain fragments generated by irradiation-induced degradation may undergo local rearrangement and self-assembly to form limited amounts of new ordered structures, the overall degree of structural order in starch still declines. These multiscale structural alterations are ultimately reflected at the microscopic level as pronounced surface etching effects on starch granules, including the formation of cracks, wrinkles, scratches, and pores [19], accompanied by disruption of internal growth-ring structures [15] and weakening or even disappearance of Maltese cross birefringence signals [86].

Within the irradiation dose range permitted for food applications, ionizing irradiation generally does not cause severe surface damage to rice starch granules. However, once the irradiation dose exceeds a certain threshold, the particle size of rice starch decreases significantly [87], while obvious surface cracks begin to appear [19]. Similar phenomena have also been reported for other A-type starches, including buckwheat starch [88,89] and corn starch [90,91], both of which exhibited varying degrees of surface disruption and pore formation after ionizing irradiation treatment.

Importantly, the irradiation-induced microstructural alterations of starch are not limited to superficial morphological changes such as cracks, pores, and fissures. These structural modifications further influence the functional properties of starch systems. Previous studies have demonstrated that hydrothermal regulation of starch granule structure can induce the formation of internal channels through amylose leaching, thereby increasing reactive sites and significantly enhancing esterification efficiency [67]. Similarly, structural changes induced by ionizing irradiation, including granule disruption and molecular degradation, are ultimately reflected in key functional properties of starch, such as gelatinization behavior, rheological characteristics, gel performance, digestibility, and chemical modification efficiency. These effects will be systematically discussed and reviewed in detail in Section 4.

#### 3.2.4. Differences Among Irradiation Types on the Multiscale Structure of Starch

Here, we summarize and integrate comparative studies on starch modification by different ionizing irradiation technologies. Considering the current limitations in the available literature, the discussion further incorporates the relationships among fine molecular structure, lamellar organization, multiscale structural evolution, and functional properties of starch. Particular emphasis is placed on elucidating the distinct mechanisms by which different ionizing irradiation technologies induce glycosidic bond cleavage, molecular degradation, lamellar disassembly, crystalline disruption, and microstructural reconstruction.

Substantial differences have been reported between EBI and X-ray irradiation in their regulation of starch multiscale structures. Importantly, the crystalline type of starch also influences its sensitivity to these irradiation treatments. Potato starch, which exhibits a B-type crystalline structure, possesses a relatively loose molecular arrangement [92], whereas lentil starch with a C-type crystalline structure has a more compact architecture [74]. At the molecular scale, EBI exerts more pronounced effects on the long amylopectin chains (B_3_ chains). Irradiation preferentially increases the proportion of medium and short chains while causing relatively mild degradation of high-molecular-weight fractions. In some cases, low-dose EBI may even induce limited molecular cross-linking. In contrast, X-ray irradiation exhibits a more uniform degradation effect on medium- and long-chain starch segments, leading to a more pronounced increase in short A chains and a greater reduction in high-molecular-weight components.

More specifically, EBI preferentially cleaves B3 long chains of amylopectin in both B-type potato starch and C-type lentil starch, converting them into medium and short chains. Under low-dose conditions, EBI can also induce slight molecular cross-linking in lentil starch, resulting in a small increase in molecular weight, while the overall degradation effect remains relatively weak [35]. In addition, EBI continuously increases the apparent amylose content in potato starch systems. By comparison, X-ray irradiation exhibits more uniform, deeper, and more extensive chain-scission characteristics, simultaneously degrading medium and long chains and generating a larger proportion of A-type short chains. The degradation of high-molecular-weight fractions caused by X-ray irradiation is substantially stronger than that induced by EBI [93], particularly in C-type lentil starch. Furthermore, special treatment conditions such as vacuum environments can further enhance the molecular degradation effects of X-ray irradiation, whereas such conditions appear unfavorable for starch degradation induced by EBI [35].

At the supramolecular scale, neither irradiation technology alters the crystalline polymorph of starch, but both exhibit distinct effects on crystallinity and short-range molecular order. For the relatively loose B-type potato starch, X-ray irradiation causes greater reductions in crystallinity and short-range order ratios (R_1047/1022_) than EBI across the entire dose range [93]. In contrast, for the more compact C-type lentil starch, the effects exhibit clear dose dependence. At low doses, EBI produces more pronounced disruption of crystalline structures, whereas under high-dose and vacuum conditions, X-ray irradiation shows stronger destructive effects on crystalline regions and short-range ordered structures.

At the microscopic scale, EBI and X-ray irradiation differ fundamentally in both their action regions and modes of structural disruption. EBI, as a particle-beam irradiation technology [11], exerts strong surface etching effects on both B-type potato starch and C-type lentil starch, while exerting relatively limited influence on internal growth rings and hilum structures. In contrast, X-rays are photon-based irradiation [94], producing more moderate and uniform surface damage while penetrating deeper into starch granules. Consequently, X-ray irradiation causes blurring of growth rings and increases the formation of internal pores and channels within granules. Studies on lentil starch further demonstrated that vacuum conditions amplify these differences: vacuum treatment intensifies surface damage caused by EBI while markedly enhancing the destructive effects of X-rays on internal structures, rendering C-type lentil starch substantially more sensitive to X-ray irradiation than to EBI under vacuum conditions. Overall, EBI is characterized by preferential surface-layer effects, relatively mild and controllable modification, and high efficiency at low doses, whereas X-ray irradiation is characterized by deep penetration, uniform chain scission, and stronger effects at high doses. Moreover, the denser the starch crystalline structure, the more pronounced the technological differences between the two irradiation methods become.

Although EBI, X-ray irradiation, and γ-rays all belong to the category of ionizing irradiation technologies, they differ fundamentally in energy-generation mechanisms, penetration depth, spatial energy distribution, and free-radical generation efficiency. EBI, as a particle-beam irradiation technology, has relatively shallow penetration depth and concentrates energy mainly at the granule surface. In contrast, X-rays and γ-rays transfer energy in the form of photons, exhibiting stronger and more uniform penetration capabilities, higher free-radical generation efficiency, and more homogeneous treatment effects [35]. These intrinsic differences are likely responsible for the distinct multiscale structural evolution patterns observed in starch, including differences in fine molecular structure, lamellar ordering, supramolecular crystallinity, and microstructural morphology.

At present, studies directly comparing the modification effects of all three ionizing irradiation technologies within a unified experimental framework remain limited. Existing research has mainly focused on EBI and X-ray irradiation, both of which are non-radioactive irradiation technologies widely applied in food processing, whereas systematic comparative studies involving γ-rays are relatively scarce. Furthermore, most comparative studies on starch crystalline systems have focused on B-type potato starch and C-type lentil starch, while systematic comparative investigations on A-type starches such as rice starch remain limited. Significant research gaps therefore still exist in areas including fine molecular chain distribution, lamellar structural evolution, and multiscale structure–function relationships in irradiated A-type starch systems.

## 4. Effects of Ionizing Irradiation on the Functional Properties of Rice Starch

Following clarification of the structural evolution of starch at the molecular, supramolecular, and microscopic scales induced by ionizing irradiation, systematic analysis at the macroscopic functional level is still required to fully elucidate the structure–function relationships of irradiated rice starch and to evaluate the practical application value of the modified starch components in subsequent processing. At the macroscopic level, the functional performance of rice starch is primarily evaluated based on measurable properties such as gelatinization characteristics, rheological behavior, swelling and solubility properties, and digestion kinetics. These functional attributes directly determine the processing suitability of rice starch as a core starch-based food ingredient and strongly influence the final quality of rice products [95]. Table 2 summarizes and compares studies published over the past six years concerning the effects of ionizing irradiation on rice and rice starch.

### 4.1. Solubility and Swelling Power

Solubility and swelling power represent the soluble and insoluble hydration characteristics of starch, respectively. As summarized in Table 2, both EBI and γ-ray exert notable regulatory effects on these two parameters. Specifically, with an increasing irradiation dose, swelling power tends to decrease progressively, whereas solubility shows a corresponding increase, both exhibiting a broadly dose-dependent pattern. This opposing behaviour can be attributed to two concurrent antagonistic effects. The increase in solubility is primarily associated with irradiation-induced chain scission and crystalline disruption: degradation generates a greater proportion of short chain fragments, which facilitates the leaching of soluble components while simultaneously exposing a larger number of hydrophilic groups [96,97]. In contrast, the decrease in swelling power results from the loss of granular structural integrity and the reduction in amylopectin molecular weight, which compromise the ability of the starch gel network to retain water upon gelatinization [98]. It should be noted, however, that the extent of these changes is influenced by multiple factors, including irradiation source type, dose range, and rice variety; therefore, the dose response relationships reported in current studies should be interpreted as general trends rather than fixed constants. Furthermore, a conceptual distinction should be made between swelling power and water-holding capacity: although both are related to starch–water interactions, water holding capacity is additionally governed by gel network continuity, polymer–polymer interactions, and the presence of low molecular weight fragments.

Swelling power, solubility, and water absorption properties are critical determinants of the eating quality of rice and rice-based products. The irradiation-induced changes in hydration behavior directly influence the texture and sensory quality of cooked rice, the viscosity of soups and starch dispersions, the smoothness and elasticity of rice noodles, as well as the compactness and surface smoothness of rice buns and rice bread crusts. More broadly, these changes ultimately affect the overall texture and palatability of rice-based foods.

It should also be noted that most existing studies consistently report a reduction in starch swelling power after irradiation treatment, indicating that ionizing irradiation alone, within practical food-processing dose ranges, is insufficient for producing highly water-retentive amylopectin-rich starches tailored for specific processing applications. Therefore, if the objective is to prepare starches with enhanced swelling capacity, further optimization of processing conditions and combined modification strategies will still be necessary.

**Table 2:** Effects of different ionizing irradiations on processing and digestive properties of rice starch.

Irradiation	Dose (kGy)	S/SP		Gelatinization Properties						Rheological/Textural				Digestibility		Reference
		S	SP	WH	GT	ΔH	PV	FV	SB/BD	G′	G″	Hd	Ad	RDS	SDS + RS	
EBI	0/1/2/3/4	/	/	/	/	/	↓	↓	/	/	/	↓	↓	/	/	[16]
	0/1/2/4	/	/	↑	↓	↓	/	/	/	/	/	↓	↑	/	/	[17]
	0/1/2/4	/	/	↑	↓↓	/	/	/	/	/	/	↓	↑	/	/	[21]
	2/4/6	↑	↑	/	↓	↓	↓	↓	↓	/	/	/	/	↑	↓	[99]
	0/1/2/3/4	↑	↓	/	↓	↓	↓	↓	↓	/	/	↑↓	↑↓	↓↑	↑↓	[100]
	0/1/3/5	/	/	/	/	/	/	/	/	/	/	↓	↑	/	/	[101]
γ-ray	1/2/5/10	/	/	↑↓	↓	/	/	/	/	/	/	/	/	/	/	[56]
	0/5/10/15	↑	↑↓	↑↓	↓	/	↓	↓	↓	/	/	/	/	/	/	[102]
	2.5/5.5/7	↑	↓	/	↑	↓	↓↓	↓↓	↓↓	/	/	/	/	/	/	[103]

/, not determined; ↑, increase; ↓, decrease; ↓↓, significant decrease; ↓↑, decreased first and then increased; ↑↓, increased first and then decreased. S, solubility; SP, swelling power; WH, water holding capacity; GT, gelatinization temperature; ΔH, gelatinization enthalpy; PV, peak viscosity; FV, final viscosity; SB, setback value; BD, breakdown value; G′, Storage modulus; G″, loss modulus; Hd: Hardness; Ad: Adhesiveness; RDS, rapidly digestible starch; SDS, slowly digestible starch; RS, resistant starch.

### 4.2. Gelatinization and Rheological Properties

Ionizing irradiation can effectively regulate the gelatinization characteristics and rheological behavior of rice starch. As summarized in Table 2, irradiation treatment significantly reduces the peak viscosity (PV), final viscosity (FV), and setback value (SB) of rice starch. As mentioned previously, ionizing irradiation induces starch degradation, leading to a reduction in long chain and high molecular weight components. On one hand, the shortening of molecular chains weakens interchain entanglement, resulting in a decrease in starch paste viscosity [11]; on the other hand, the low molecular weight short chain fragments generated through depolymerization and debranching can interfere with the ordered reassociation of starch molecules, suppress recrystallization, and reduce moisture migration, thereby retarding the retrogradation process of starch to a certain extent [104].

Existing studies further demonstrate that electron beam irradiation, γ-rays, and X-ray irradiation exhibit broadly similar modification patterns toward starch. Although these treatments generally do not alter the crystalline polymorph of starch, they significantly reduce crystallinity and gelatinization enthalpy (ΔH), and in most cases lower the gelatinization temperature (GT). Such characteristics are advantageous for rice products requiring reduced processing temperatures, including parboiled rice [105] and instant rice porridge products [1].

In addition, irradiation treatment decreases both the storage modulus (G′) and loss modulus (G″), resulting in changing gel strength and enhanced flowability, while simultaneously lowering syneresis and improving freeze–thaw stability. These influences in rheological properties make irradiated rice starch more suitable for the industrial production of convenience rice, rice noodles, vermicelli, and frozen rice-based products. Irradiation-modified starch can significantly enhance textural stability during heating, shearing, and freeze–thaw processing [106,107], while reducing quality defects such as water separation [104] and cracking [75].

### 4.3. Digestibility Properties

The digestibility of starch is one of the most critical factors determining the glycemic index (GI) and nutritional value of staple food products. Similar to starches from other botanical sources, the proportions of rapidly digestible starch (RDS) and digestion-resistant fractions, including slowly digestible starch (SDS) and resistant starch (RS), in rice starch are highly dependent on its multiscale structural organization. Comprehensive in vitro digestion studies on rice starch and starches from various other sources [19,35,76,77,81,82,93,104,108] have demonstrated that ionizing irradiation, within an appropriate dose range, can significantly improve the digestibility properties of rice starch by regulating crystalline structure, double-helical ordering, and lamellar organization. These modifications are typically reflected by increased resistant starch (RS) content and reduced digestion rates [19].

Native rice starch exhibits a typical A-type crystalline pattern in its XRD diffractogram [4,17,19]. Klostermann et al. [99] reported that A type crystals undergo slow hydrolysis through a “crystal-by-crystal” mechanism during digestion; however, this does not imply that the A type crystalline structure is the dominant factor determining the formation of resistant fractions. A comprehensive interpretation of the regulatory effects of ionizing radiation on anti-digestive components requires consideration of systematic changes in multiscale structures.

From the molecular and supramolecular perspectives, the fundamental mechanism by which ionizing radiation influences rice starch digestibility involves disruption of crystalline regions, dissociation of double helical structures, and molecular chain rearrangement. These structural alterations collectively modify the efficiency of interaction between starch substrates and digestive enzymes. Specifically, irradiation typically disrupts crystalline ordered regions and double helices, exposing more enzyme accessible sites and thereby increasing the contact efficiency between enzymes and substrates. Simultaneously, irradiation generates substantial amounts of short chain molecules, which can trigger molecular rearrangement, chain reassociation, or recrystallization, forming new ordered structures resistant to enzymatic hydrolysis [73]. In conjunction with the changes in R_1047/1022_ and RC ordered structural parameters reported in other ionizing radiation studies summarized in Table 1, it is evident that several studies have observed varying degrees of increased ordered structures in starch following irradiation treatment.

The effect of irradiation dose should not be overlooked. Low dose irradiation exerts relatively mild effects, causing only slight chain scission and local structural perturbations, yet it can promote the rearrangement and aggregation of short-chain amylose molecules, thereby increasing the total content of slowly digestible starch (SDS) and resistant starch (RS). In contrast, high-dose irradiation eventually leads to extensive starch degradation, enhancing enzyme accessibility and making the starch more susceptible to hydrolysis [19,109], which is reflected in a marked increase in rapidly digestible starch (RDS) content.

In general, the effect of ionizing radiation on rice starch digestibility is not limited to merely increasing the proportion of rapidly digestible fractions. From a theoretical perspective, by rationally controlling the irradiation energy transfer efficiency during processing, it is possible to achieve targeted modulation of starch digestibility properties. This approach could, in principle, offer a potential technical strategy for developing rice-based staple products with a lower glycemic index, slower digestion rates, and improved glycaemic responses. However, it must be emphasised that this concept is still in its preliminary stages and remains theoretical at present. Although existing research evidence provides some useful reference, the specific mechanisms and pathways underlying the formation of irradiation-induced resistant starch fractions have not yet been systematically or thoroughly investigated or explained. In the absence of sufficient mechanistic understanding and nutritional evaluation, the notion of achieving “precisely targeted” production of slowly digestible or resistant starch through irradiation alone at an industrial level can only be regarded as a future direction, rather than a mature and immediately applicable solution. Considerable further research is still needed to elucidate the intrinsic structure function mechanisms and to establish quantitative models that link single irradiation doses, cumulative irradiation doses, and other processing parameters to starch digestibility behaviour.

### 4.4. Multiscale Structure-Function Relationship of Rice Starch

The multiscale structure of starch is closely associated with its macroscopic functional properties, forming the fundamental theoretical basis for understanding and regulating the quality of rice-based products. At the molecular scale, chain-length distribution, molecular weight, and the amylose-to-amylopectin ratio collectively determine starch solubility, swelling power, gelatinization viscosity, and rheological behavior. In particular, the branching degree and chain-length distribution of amylopectin are directly related to starch swelling characteristics [110]. Generally, the longer the molecular chains and the denser the branching structure, the more compact the internal organization of starch granules becomes, resulting in weaker dissolution and hydration capacity under hydrothermal conditions.

**Figure 2:**
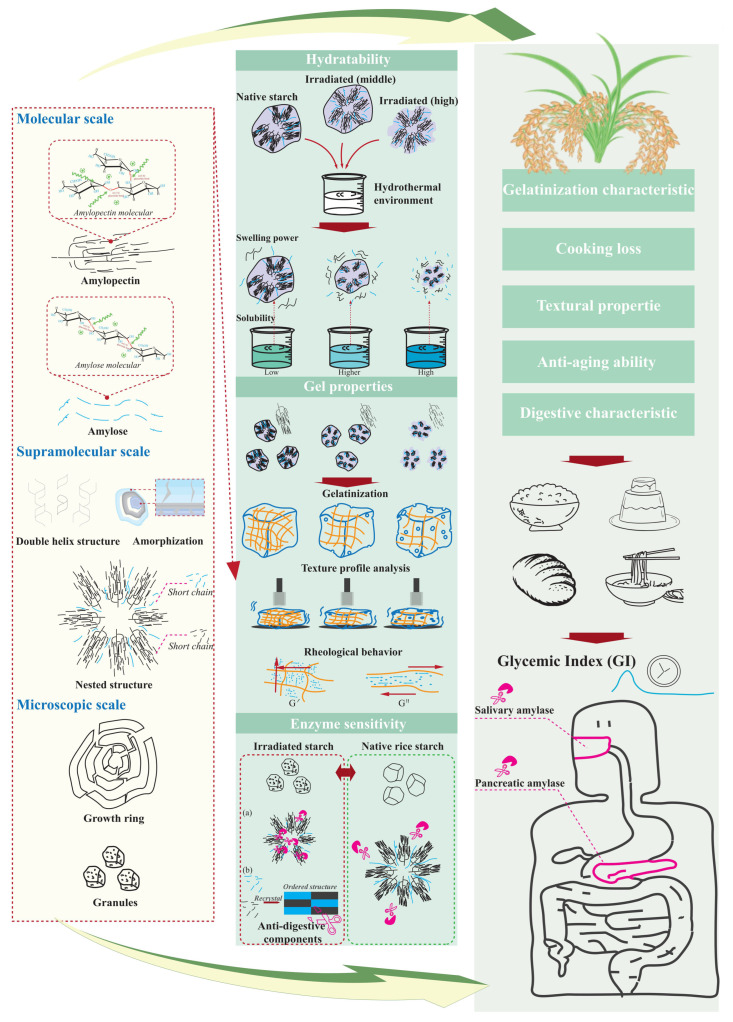
Schematic diagram of ionizing irradiation-induced multiscale structural evolution of rice starch and its correlation with processing and nutritional properties of rice and rice products. From left to right: regulatory mechanism of ionizing irradiation on starch multiscale structure; effects of structural changes on hydration, gelation, enzyme sensitivity and other processing properties, (a) Enzymatic accessibility of damaged starch structures after irradiation treatment; (b) Recrystallization of short starch chains generated by irradiation and its resistance to enzymatic hydrolysis; key processing evaluation indices of rice raw materials and major digestive sites of rice starch in the human digestive system.

At the supramolecular scale, the swelling behavior of starch is essentially governed by the strength of intermolecular interactions between the crystalline and amorphous regions, which primarily originate from hydrogen bonding among amylopectin side chains [111,112]. It is important to distinguish clearly between two related but distinct parameters: swelling power, which reflects the extent of granule swelling upon heating in excess water, and water-holding capacity, which denotes the ability of a starch gel or granule network to retain water against an external force. These two properties are modulated by different factors. Swelling power is largely determined by granule structural integrity and the molecular characteristics of amylopectin, whereas water holding capacity is additionally influenced by gel network continuity, polymer-polymer interactions, and the presence of low molecular weight degradation fragments. In general, starches with higher amylopectin content and more intact ordered structures tend to be more resistant to complete swelling during gelatinization [113]. Based on previous findings [67], a “nested structural model” (Figure 2) can be adopted to theoretically describe the architecture of rice starch: highly branched, high molecular weight amylopectin forms a dense structural framework, while amylose molecules occupy the interstitial regions and contribute to the stabilization of the aggregated structure. Starch granules characterized by extensive branching, large molecular weight, and a high proportion of long chains generally exhibit a more compact molecular packing, which tends to restrict amylose leaching and limit initial hydration at the granule surface under hydrothermal conditions. Consequently, such starches often display lower swelling power under conventional heating regimes. However, their water-holding capacity does not necessarily covary with swelling power; instead, it is simultaneously influenced by multiple factors, including gel network integrity, molecular chain reassociation behaviour, and the specific measurement conditions employed.

At the supramolecular level, crystallinity, double-helical ordering, and lamellar regularity directly determine swelling behavior, gelatinization enthalpy, thermal stability, and digestion rate. At the microscopic level, starch granule integrity, internal pore structure, and surface morphology significantly influence water absorption kinetics, enzymatic accessibility, and the final textural properties of starch-based products. Ionizing irradiation enables coordinated multiscale regulation ranging from molecular depolymerization and chain scission to supramolecular crystalline disruption and microstructural pore formation. On the one hand, irradiation breaks long molecular chains, reduces molecular weight and branching density, and weakens intermolecular hydrogen bonding. On the other hand, it disrupts the ordered arrangement of crystalline regions, decreases crystallinity and double-helical content, and simultaneously generates microcracks and pores on granule surfaces.

Through this coordinated regulation of molecular-chain structure, supramolecular organization, and starch granule microstructure, ionizing irradiation can significantly alter macroscopic functional properties of rice starch, including solubility, swelling power, gelatinization behavior, and rheological characteristics. Although these macroscopic functional changes may appear independent, they are in fact closely interconnected through hierarchical structural responses spanning the molecular, supramolecular, and microscopic scales.

### 4.5. Dose-Dependent Analysis of Structural Evolution and Functional Properties

Based on the data compiled in Table 2 and the dose-related findings discussed in Section 3.2, Section 4.1, Section 4.2, Section 4.3 and Section 4.4, comparable dose-dependent trends can be derived. Although the extent of structural changes in irradiated starch may vary considerably depending on rice cultivar, irradiation type, and processing conditions, the overall pattern can still be generalized into a “three-tier dose regime.” To consolidate the correspondences between structural changes and functional responses of rice starch across different dose levels, this analytical framework is briefly summarized in Table 3.

At low doses (typically ≤ 5 kGy), the perturbation to the multiscale structure of starch is relatively limited. As detailed in Table 2, rice starch subjected to EBI or γ-ray irradiation at this dose range exhibits only minor changes in molecular size and granule integrity [16,17,22]. The reductions in PV and FV are modest, and although solubility increases slightly [114], the overall magnitude remains small. Functionally, the most notable change is the retardation of retrogradation, which is primarily attributed to the limited generation of short-chain fragments that interfere with chain reassociation [17,105].

Medium doses (approximately 5–10 kGy) induce more pronounced degradation effects. Studies on EBI-treated rice starch consistently show significant depolymerization of amylopectin long chains, accompanied by marked reductions in RC and the short-range order parameter R_1047/1022_ [19,114]. These supramolecular disruptions further manifest as substantially lowered PV, FV, and setback values, together with increased solubility and decreased swelling power [109,114]. Rheologically, the G′ decreases, reflecting a weakened gel network [17]. Regarding digestive properties, some studies have associated this dose range with increased levels of SDS and RS fractions, possibly due to molecular rearrangement of degradation fragments under specific conditions [19]; however, the exact magnitude of these changes is highly dependent on cultivar and irradiation conditions and should be evaluated on a case-by-case basis.

At high doses (>10 kGy), the degradative effects on starch molecules become drastic. Chain scission intensifies continuously, leading to the accumulation of substantial low molecular weight components and near-complete disruption of crystalline order, accompanied by a sharp decline in RC and pronounced surface cracking and pore formation [19,73]. Functionally, gel network strength and water-holding capacity are severely compromised, while solubility becomes excessively high and cooking loss increases markedly [57,115]. Of particular concern, under refrigerated or freeze–thaw conditions, the abundant short chain fragments may paradoxically promote recrystallization, accelerating quality deterioration [116,117,118]. Consequently, high-dose irradiation is generally not recommended for intact granule or gel-type products.

## 5. Effects on Rice Product Quality via Starch Multiscale Structure

### 5.1. High-Moisture Rice Products

With the continuous improvement of cold-chain logistics and modern supply-chain systems, fresh and high-moisture rice products have become increasingly popular in the food market [119]. In southeastern China, fresh rice noodles, rice cakes, and glutinous rice cakes are among the most representative fresh rice-based products, highly favored by consumers because of their soft texture, smooth mouthfeel, moderate elasticity, and appropriate stickiness. The core quality requirements for these products include softness, moderate elasticity, smoothness without stickiness, resistance to retrogradation, and stable shelf life. For rice noodles specifically, good cooking tolerance, soaking resistance, and low breakage rates are also essential.

These products are generally characterized by high moisture content and diverse processing conditions. Fresh rice noodles are typically produced under hydrothermal conditions, rice cakes are mainly prepared by steaming, while glutinous rice cakes often involve both hydrothermal and steam-based processing. Owing to the diversity of processing methods, consumption scenarios, and quality requirements, fresh rice products place high demands on the processing adaptability, texture stability, and sensory acceptability of raw materials. As the major component of rice and the primary structural framework of rice-product gel networks, the structural characteristics of rice starch directly determine processing performance and final product quality.

From the perspective of quality control, the desirable processing performance, eating quality, and storage stability of fresh high-moisture rice products depend largely on the hydration capacity and water-holding capacity of rice starch, which are closely associated with granule integrity and the average chain length of amylopectin [120]. No tably, the irradiation dose remains a factor that cannot be overlooked. In general, within the safety limits permitted for food applications, starches subjected to higher radiation doses—owing to their increased proportion of low-molecular-weight fractions and short chains—are more suitable for food systems that exhibit emulsion-like characteristics [11]. For products requiring a relatively intact gel network, such as rice cakes, excessive degradation beyond a certain dose threshold may lead to over-softening and structural collapse, ultimately compromising product integrity.

In line with the earlier summary of ionizing radiation effects on starches from other botanical sources, it is evident that electron beam irradiation, X-rays, and gamma rays exert a dominant degradative action on both amylose and amylopectin, particularly at high doses. Consequently, irradiated rice starch, with its increased short-chain fraction and reduced amylopectin molecular weight, tends to exhibit greater cooking loss, diminished gel integrity, and reduced water-holding capacity during processing. The extent of these changes is also influenced by rice variety, given that waxy rice contains a substantially higher proportion of amylopectin compared to japonica and indica rice. Such modifications may induce undesirable stickiness and turbidity in the cooking water during the production and processing of rice noodles and rice cakes, thereby adversely affecting the sensory quality of the final products.

For food systems that benefit from a higher proportion of low molecular weight components, however, irradiation-induced debranching of amylopectin, degradation of amylose, and generation of additional short chains may actually confer processing advantages. For example, the typical production of fermented rice cakes involves a fermentation step of rice batter, which like rice pudding systems [9], shares essential structural features with pickering emulsions stabilized by starch particles, a starch–gas–water three-phase system in which starch acts as an emulsifier [121]. Starch molecular weight plays a critical role in modulating the stability of Pickering emulsions: lower molecular weight is associated with stronger emulsifying ability of starch particles and greater emulsion stability [122]. Ionizing radiation significantly reduces starch molecular weight and chain length, and complexes formed between irradiated rice starch and proteins have already been demonstrated to possess favourable emulsifying properties [123]. Based on these findings, it may be inferred that the addition of irradiated rice starch enriched with low molecular weight degradation products could improve gas-holding capacity in fermented rice cake systems, resulting in more uniform pore structures within the starch gel following thermal processing. In rice pudding, similar modifications may further enhance smoothness and creaminess. As summarized in Table 2, ionizing radiation significantly decreases the G′ value of rice starch gels, indicating a reduction in gel strength; corresponding texture analysis data further confirm decreases in starch hardness. Reductions in gel strength, hardness, and elasticity are advantageous for fresh rice noodles, glutinous rice cakes, and other rice products that require a softer texture.

Notably, the free radicals generated during electron beam irradiation [56] and the subsequent starch depolymerization [104] have been shown to interfere with intermolecular hydrogen bonding or generate short-chain fragments, thereby hindering the ordered reassociation of starch molecular chains and effectively retarding starch gel retrogradation while reducing moisture migration under ambient conditions. For room-temperature storage, this effect undoubtedly helps delay quality deterioration of rice products caused by starch retrogradation. However, low-temperature storage environments, particularly those involving freeze–thaw cycles, may operate through distinctly different mechanisms. Although overall moisture migration slows at low temperatures, the formation of ice crystals and the sublimation of water under sub-zero conditions can still inflict irreversible physical damage to the starch gel network. More critically, frequent moisture migration significantly accelerates the recrystallization of starch components, especially short chain fractions [116], ultimately leading to a rapid decline in gel water-holding capacity, increased cooking loss, and compromised quality stability of rice products [117,118].

The short-chain fragments induced by irradiation exhibit a typical dose-dependent “double-edged sword” effect in retrogradation regulation: whether they exert anti-retrogradation or pro-recrystallization effects depends primarily on the synergistic interplay between irradiation dose and storage temperature. Under low-dose irradiation combined with ambient storage, the limited short chains generated interfere with intermolecular ordered packing, thereby inhibiting starch retrogradation. In contrast, under high-dose irradiation or freeze–thaw conditions, the substantial accumulation of short-chain fragments exacerbated by enhanced moisture migration and increased local concentration shifts the balance towards recrystallization by serving as nucleation sites, ultimately favouring structural deterioration.

Considering the gradient of irradiation doses: low-dose irradiation generates only a small number of short-chain fragments, which are sufficient to hinder chain ordering and suppress retrogradation under appropriate temperature conditions while preserving adequate molecular integrity to maintain the gel structure; moderate-dose irradiation presents a clear trade-off, wherein amylopectin and amylose undergo degradation, but to a lesser extent than at high doses, resulting in improvements in certain functional properties (such as emulsifying capacity and gel texture softening), albeit accompanied by some degree of textural deterioration; high-dose irradiation induces more extensive multi scale structural disruption of starch, producing abundant short chain fragments that particularly promote recrystallization under freeze–thaw conditions, while concurrently causing marked reductions in gel strength, water-holding capacity, and overall processing adaptability.

Based on the above analysis, future research on preservation technologies for fresh high-moisture rice products should carefully consider the differences between storage conditions and consumption scenarios, achieving a rational trade-off between texture improvement and freeze–thaw stability through dose optimization. Only under appropriate conditions can the modifications induced by irradiation on the multiscale structure of rice starch including enhanced solubility, altered pasting viscosity, textural softening, and improved emulsifying properties, be effectively harnessed. At the same time, a more systematic investigation into the differential effects of the three ionizing radiation technologies on the multiscale structure of rice starch, as well as how to mitigate irradiation-induced processing adaptability loss and accelerated retrogradation through integration with other modification strategies and process engineering, remains an important direction for future systematic research.

### 5.2. Low-Moisture Rice Products

Instant staple foods have evolved far beyond their early, highly standardized forms. Driven by advances in processing and packaging technologies, instant rice-based products have gradually become a major segment of the convenience food market because of their ease of preparation and consumption, while product categories continue to diversify [124]. Based on product form, low-moisture rice products can generally be classified into three categories: strand-shaped rice noodles, granular instant rice, and powdered instant rice products. It should be noted that instant rice is not conventional milled rice, but rather cooked and restructured rice produced through thermal processing and modification. Different processing technologies impart distinct quality characteristics to instant rice products, and these processing features and quality patterns have been systematically summarized in previous reviews [125,126].

Dried rice products, represented by dehydrated instant rice noodles and instant rice, are typical gel systems formed from pregelatinized starch. During processing, the starch components in rice first undergo gelatinization under hydrothermal conditions. During this process, starch molecules become fully hydrated, molecular chains are extended, and intermolecular entanglement gradually forms a continuous starch gel network [1]. Subsequent dehydration and drying accelerate water loss from the system, promoting hydrogen-bond reassociation and retrogradation rearrangement of starch molecules. As a result, the gel structure progressively solidifies and contracts, ultimately forming low-moisture dried rice noodles and instant rice matrices with dense and rigid structures [127]. Before consumption, these low-moisture dried rice products must undergo a rehydration process [128].

Unlike high-moisture fresh rice products, which are characterized by “dissolution followed by gel formation,” dried instant rice products already possess a relatively complete and stable gel network before rehydration, and mass loss mainly occurs during rehydration as soluble components leach out. Water migration during rehydration follows a gradient-driven process from the outer surface inward: water first penetrates the surface layer and then gradually diffuses through gel pores into the interior. During this process, the gel structure absorbs water, expands, and restructures, while soluble starch components progressively leach into the surrounding medium. In daily cooking practice, low-quality dried rice products often exhibit excessive leaching of soluble components during gradient rehydration, leading to increased cooking loss, excessive surface stickiness, and structural disintegration. These phenomena are closely associated with the structural stability of the gel system under severe hydrothermal conditions [129,130].

As summarized in Table 2 and discussed in the previous section on gelatinization properties, ionizing irradiation can significantly alter the molecular, supramolecular, and microscopic structures of rice starch, thereby markedly modifying its gelatinization behavior. The most prominent changes include the coordinated reduction in four key parameters: peak viscosity, final viscosity, breakdown value, and setback value. Combined with the corresponding rheological changes, the gel structure formed by irradiated rice starch appears less capable of maintaining the elastic and chewy texture required for dried rice noodles and instant rice products. However, for powdered instant rice products, these irradiation-induced processing characteristics may provide unique advantages, including low viscosity, high stability, and reduced retrogradation tendency, while also significantly lowering the required reconstitution temperature [131,132].

The reduction in peak and final viscosities can prevent excessive thickening after reconstitution, while the marked decrease in G″ contributes to a smoother and more palatable mouthfeel, thereby reducing swallowing difficulty [133]. Meanwhile, the lower breakdown value helps maintain viscosity stability during hot-water reconstitution, preventing localized thinning or lump formation and ensuring a more uniform texture. The reduction in setback value can delay post-reconstitution retrogradation, thereby preventing hardening, syneresis, and stickiness, and ultimately extending the acceptable consumption window. Beyond powdered instant rice products, irradiated rice starch with these modified properties also shows considerable potential for application in low-viscosity instant beverage products, such as gluten-free meal replacement powders [134].

## 6. Current Applications of Ionizing Irradiation Technology in Rice Processing

EBI, γ-rays, and X-ray irradiation are three representative non-thermal ionizing irradiation technologies that have been extensively studied and applied in the food industry. Their applications span multiple areas, including storage and preservation [13,135,136,137], component modification [15,42,77], and the preparation of food-grade composite materials [123].

Considering the full life-cycle of rice products from harvest to consumption, the application scenarios of ionizing irradiation can be divided into four major stages: harvesting, primary processing, deep processing, and storage/distribution. During storage and preservation, ionizing irradiation represented by EBI can achieve highly efficient microbial inactivation [12,138] and mycotoxin degradation [14,100], thereby significantly improving the safety of rice raw materials. This is particularly important for newly harvested high-moisture paddy rice [17,19], where irradiation can enhance storage stability while maintaining the sensory and nutritional quality of rice [16,22,114].

During the primary processing stage, ionizing irradiation can effectively affect the functional properties of processing by-products [139], thereby enhancing the valorization and utilization efficiency of rice by-products [101,140,141,142]. Furthermore, at the deep processing stage, different irradiation doses regulate the multiscale structural evolution of rice starch, thereby altering the processing behavior of starch and potentially influencing gelatinization properties, textural characteristics, and retrogradation behavior in a dose-dependent manner. The extent and direction of these effects, whether beneficial or detrimental, depend on the specific irradiation dose, starch variety, and processing conditions employed. However, studies directly using irradiated rice as a raw material for deep processing remain limited. Early studies, such as Sung reported [131], applied γ-irradiated rice to rice tofu products, whereas more recent research has primarily focused on the effects of EBI on rice cooking quality and eating characteristics [16,17,22,114,143]. During storage and distribution, ionizing irradiation functions similarly to postharvest preservation technologies by extending shelf life and improving food safety through efficient sterilization.

Despite these advances, the application of ionizing irradiation in the deep processing of rice products still faces several major challenges. High-dose irradiation may induce excessive aggregation of rice glutelin proteins [78] while simultaneously causing excessive degradation of starch molecular chains [19], ultimately reducing the processing suitability of rice raw materials. In addition, precise control of irradiation dose remains difficult, and variations in composition and structure among rice cultivars lead to substantial differences in irradiation response patterns, preventing the establishment of universally applicable standardized processing systems. Furthermore, limited consumer awareness and low acceptance of irradiated foods, together with the high installation and operational costs associated with certain irradiation technologies (particularly X-ray and γ-rays systems), continue to restrict the industrial-scale application of ionizing irradiation as a functional modification strategy in food processing.

## 7. Future Perspectives

Although considerable progress has been made in current research, several critical limitations persist. At the fundamental research level, the dynamic mechanisms underlying irradiation-induced multiscale structural changes in rice starch remain poorly understood. In particular, there is a notable lack of quantitative studies on the effects of irradiation penetration depth, free radical generation efficiency, and processing dosage on polysaccharide chain scission and crosslinking, as well as a shortage of mechanistic interpretations that establish dynamic correlations between in situ multiscale structural evolution and irradiation parameters. At the application level, tailored irradiation treatment models for rice varieties at different physiological states and for specific rice products have not yet been established, leaving industrial production without systematic theoretical guidance. Meanwhile, the synergistic effects of irradiation with other processing technologies, such as enzymatic hydrolysis and fermentation, remain to be further explored. With regard to irradiation facilities and their promotion, the large-scale application of irradiation technology is still hindered by factors including relatively high operational costs, insufficient process standardization, and limited consumer awareness of the safety of irradiated foods.

To promote broader application of ionizing irradiation technology in rice product processing and to provide more comprehensive theoretical support for future industrialization, subsequent research should be systematically advanced in several key directions. First, with respect to fundamental mechanisms, greater efforts should be devoted to investigating the dynamic evolution of rice starch multiscale structures during irradiation, integrating advanced in situ characterization techniques with molecular simulation approaches to elucidate the regulatory mechanisms governing chain scission and crosslinking. Second, in terms of technological innovation, precise dose-control strategies should be developed, and the combined application of irradiation with other processing methods, such as fermentation and enzymatic treatment, should be explored to further optimize irradiation parameters while ensuring food safety, thereby improving the textural quality and storage stability of rice products. Third, the application of artificial intelligence techniques, including machine learning and deep learning, should be utilized to construct more precise correlation models connecting the multiscale structure of starch with the quality attributes of rice products, thus offering both practical model references and a theoretical basis for the development of healthier rice-based foods. In addition, greater investment should be directed toward industrial translation, including the development of low-cost, modular irradiation equipment adaptable to diverse processing scenarios, as well as the construction of standardized systems covering raw material selection, dose regulation, and process parameter optimization. Meanwhile, public education on the safety of irradiated foods should be strengthened to bridge the knowledge gap between consumers and emerging food technologies. Finally, future research should further extend the application of irradiation technology to functional rice products, such as resistant starch-based products and low-glycemic-index foods, with particular emphasis on elucidating the formation mechanisms and nutritional characteristics of digestion-resistant components across different structural scales. Also, it should be noted that the main perspective of this review is grounded in the multiscale structural changes of starch, and the studies summarized are predominantly based on purified starch model systems. In reality, rice-based foods are complex multi-component matrices in which starch, proteins, lipids, and dietary fibers coexist and interact during processing. Such interactions among components can significantly influence the effectiveness of irradiation on overall product quality. Therefore, future research should further refine the full chain mechanistic relationship connecting irradiation, starch structural evolution, and functional properties, with particular emphasis on elucidating the roles of non-starch components and their interaction patterns with starch under irradiation conditions.

## 8. Conclusions

The effects of ionizing radiation on the multiscale structure of rice starch generally follow similar trends to those observed for starches from other botanical sources. However, marked differences in starch composition, particularly the amylose/amylopectin ratio, exist among rice varieties such as waxy, japonica, and indica, leading to distinct irradiation responses under identical treatment conditions. Within food safety dose limits, electron beam, γ-ray, and X-ray irradiation all promote degradation of long starch chains and branch structures, facilitating the breakdown of high-molecular-weight components into low-molecular-weight fractions. The extent of degradation and debranching is governed not only by the intrinsic composition of rice but also by treatment parameters including irradiation source type, absorbed dose, and moisture content. Beyond a variety-specific critical dose threshold, starch undergoes more pronounced structural alterations, including surface cracking, particle size reduction, growth ring disruption, and loss of Maltese cross birefringence. These changes across molecular, supramolecular, micro-, and macro-scales differentially modulate pasting, rheological, textural, and digestive properties, thereby establishing a coherent irradiation–structure–function relationship whose intensity depends on the interplay between processing conditions and raw material characteristics.

Building on this framework, the present review has comprehensively consolidated current knowledge on irradiation-induced structural modulation of rice starch, clarified the distinct roles of different irradiation sources and dose regimes, and elucidated the underlying mechanisms linking structural evolution to product quality. Key technical bottlenecks and optimization strategies have also been critically assessed. Looking forward, the full-chain relationship among irradiation parameters, structural dynamics, and functional outcomes warrants further refinement, with particular emphasis on integrating precise dose control with combinatorial modification approaches to address the inherent limitations of single-treatment strategies. Ionizing irradiation holds considerable promise for the deep processing of rice products; by establishing product-specific optimal dose windows, it may be feasible to concurrently extend shelf life and enhance quality attributes. As process standardization matures and consumer acceptance improves, ionizing irradiation is poised to offer a viable technological pathway toward green, efficient, and high-value rice processing, facilitating the broader transition from primary processing to advanced manufacturing in the agricultural sector.

## Figures and Tables

**Table 1:** The effects of different ionizing irradiations on the multiscale structures of starch from different sources.

Irradiation	Starch	Crystal Type	Molecular				Supramolecular		Microscopic			Reference
			CLD	Mw	AC	Functional Groups	R_1047/1022_	RC	Growth Ring	Granule Morphology	Maltese Cross	
EBI	Rice	A	L↓S↑	↓	↓	-	/	↓	/	D	/	[18]
	Wheat	AB	L↓S↑	↓	/	-OH↑	↓	↓	/	-	/	[55]
	Potato	B	L↓S↑	↓↓	/	/	↓	↓	B	-	B	[72]
			/	/	↑	-	↑↓	↑↓	/	-	B	[74]
			L↓S↑	↓	/	-	↑↓	↓	-	D	-	[75]
			/	↓	/	-COO-↑ -OH↑	/	↑↓	/	/	/	[76]
			L↓S↑	↓	/	-	↓	↑	-	-	-	[77]
	Corn	A	L↓S↑	↓	↓	-OH↑	↓↑	↓	/	-	/	[52]
			L↓S↑	↓	↑↑	-OH↑	↓	↓	/	D	/	[78]
			L↓S↑	↓	-	-	/	↓	/	-	/	[79]
	Oat	A	L↓S↑	↓↓	/	-	↓	↓	-	-	-	[80]
	Chestnut	C	L↓S↑	↓↓	↓	-OH↓	↓	↓	/	-	/	[81]
X-ray	Lentil	C	L↓S↑	↓↓	↓↑	-OH↑	↓	↓	-	D	/	[34]
	Potato	B	/	/	↑↑	-OH↑	↑↓	↑↓	/	A	B	[74]
	Wheat	AB	L↓S↑	↓↓	↓	-OH↑	↑	↑	B	D	/	[82]
γ-ray	Chickpea	C	/	/	↓	-	↓	↓↑	/	D	/	[83]
	Faba bean	C	L↓S↑	↓	↓	-	↓	↓	/	D	/	[84]
	Wheat	AB	L↓S↑	↓	/	-	-	↓↑	/	-	/	[85]

L↓S↑, Long-chain fraction decreased while short-chain fraction increased; -, no significant change; /, not determined; ↑, increase; ↓, decrease; ↓↓, significant decrease; ↓↑, decreased first and then increased; ↑↓, increased first and then decreased. B: Blurred; D: Damage; A: Aggregate; CLD: chain length distribution; Mw: molecular weight; AC: amylose content; R_1047/1022_: ratio of short-range ordered structure; RC: relative crystallinity.

**Table 3:** Dose-dependent structural and functional responses.

Dose Range	Structural Changes	Functional Changes	References
Low (≤5 kGy)	RC ↓	PV/FV ↓, S ↑; retrogradation ↓	[16,17,22,104,114]
Medium (5–10 kGy)	Debranching (L→S) ↑ RC & R_1047/1022_ ↓↓ surface etching ↑	PV/FV/SB/G′ ↓↓; S ↑↑/SP ↓↓	[19,73,109,114]
High (>10 kGy)	Debranching (L→S) ↑↑ RC & R_1047/1022_ ↓↓↓ surface etching ↑↑	WH ↓↓↓; S ↑↑↑; recrystallization ↑↑(freeze–thaw)	[19,57,73,115]

RC, relative crystallinity; S, solubility; SP, swelling power; WH, water holding capacity; PV, peak viscosity; FV, final viscosity; SB, setback value; G′, Storage modulus; ↑, slightly increase; ↓, slightly decrease; ↑↑, increase; ↓↓, decrease; ↑↑↑, significant increase; ↓↓↓, significant decrease.

## Data Availability

The original contributions presented in this study are included in the article. Further inquiries can be directed to the corresponding author.
